# Supplementary figures and images for: Efficacy and safety of intracavitary electrocardiography-guided peripherally inserted central catheters in pediatric patients: a systematic review and meta-analysis
Source: PeerJ. 2024 Oct 8;12:e18274. doi: 10.7717/peerj.18274 (PMC11468838; doi:10.7717/peerj.18274)

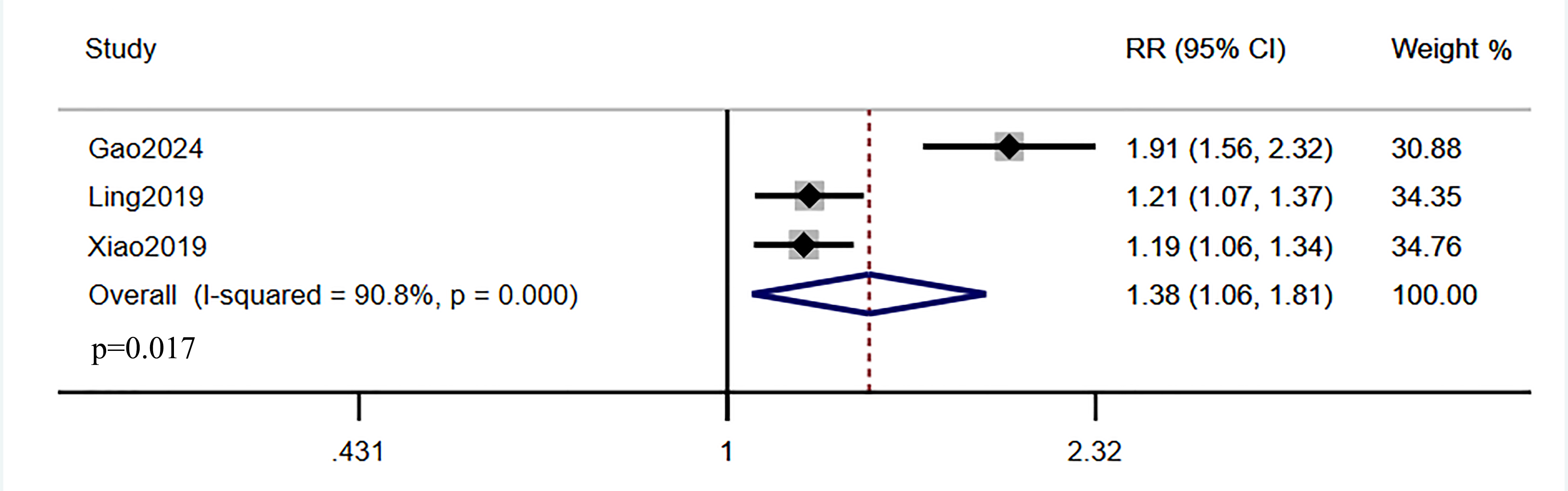

Supplement: Supplemental Information 1 [file peerj-12-18274-s001.png]

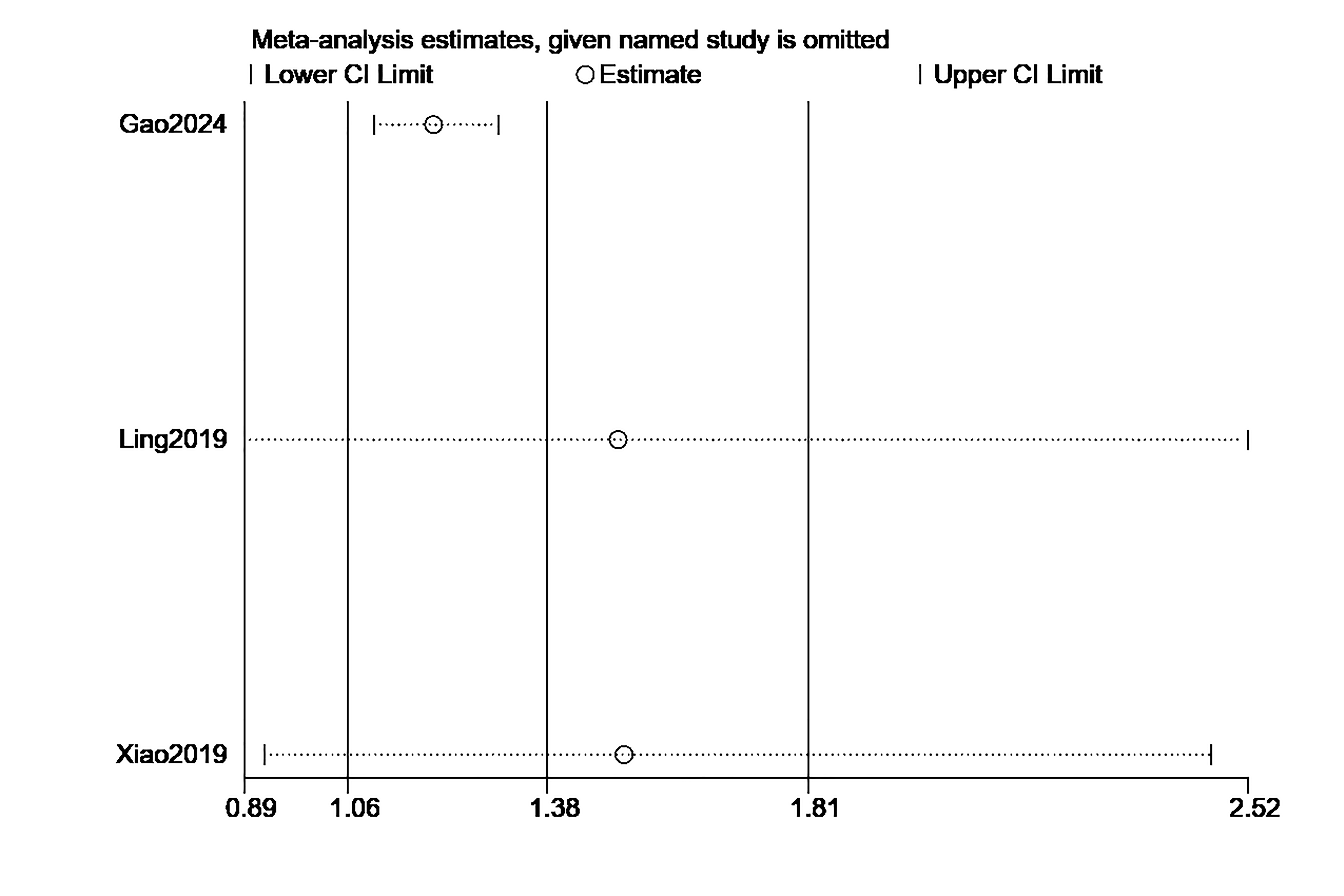

Supplement: Supplemental Information 2 [file peerj-12-18274-s002.png]

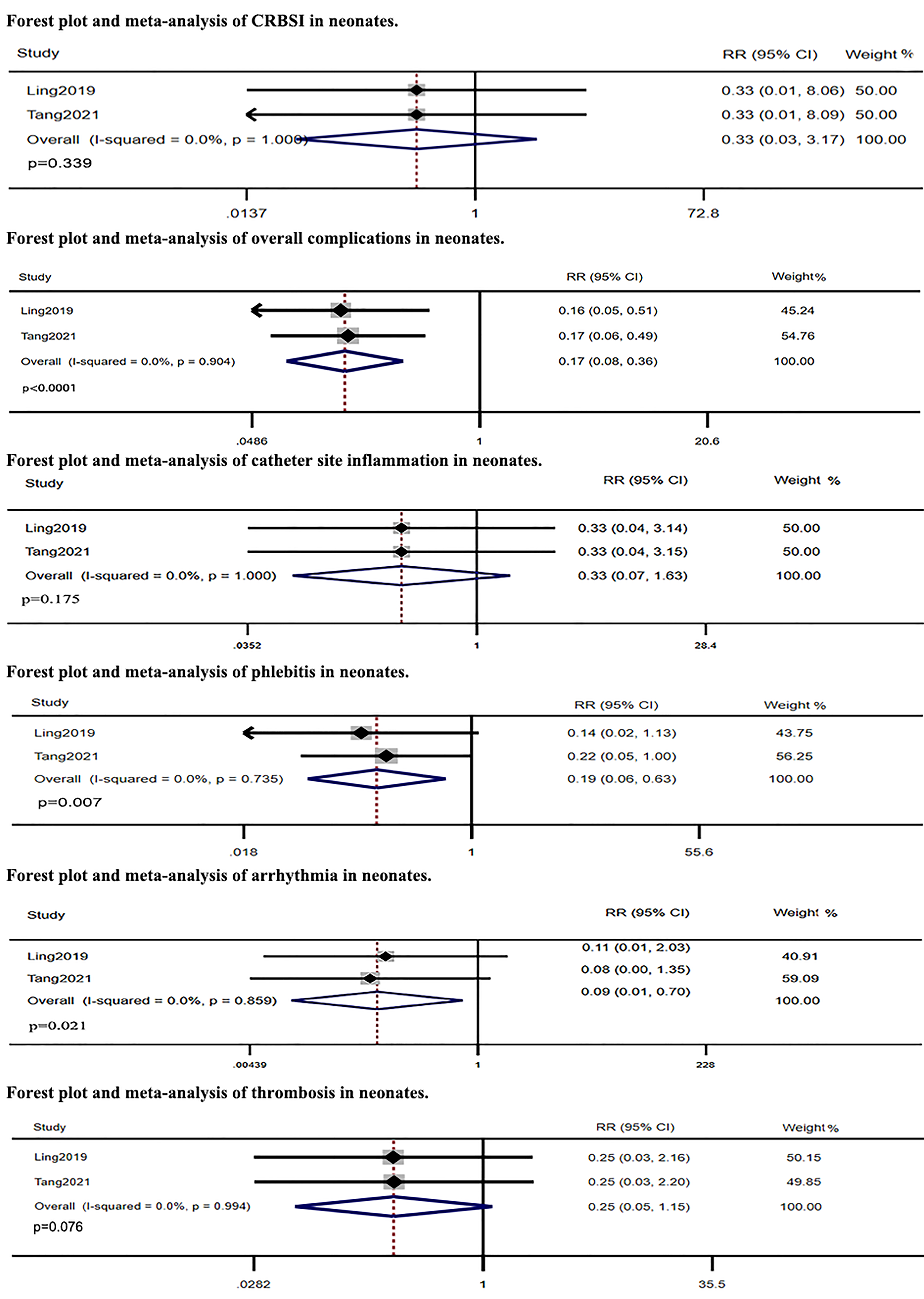

Supplement: Supplemental Information 4 [file peerj-12-18274-s004.png]
