# Supplementary material for: Efficacy and safety of intracavitary electrocardiography-guided peripherally inserted central catheters in pediatric patients: a systematic review and meta-analysis
Source: PeerJ. 2024 Oct 8;12:e18274. doi: 10.7717/peerj.18274 (PMC11468838; doi:10.7717/peerj.18274)
Supplement: Supplemental Information 3 [file peerj-12-18274-s003.docx]

**For Pubmed databases**

**ID**  **Search**

#1 intracavitary electrocardiography [All fields]

#2 IC-ECG [All fields]

#3 electrocardiography [MeSH terms]

#4 OR #1-3

#5 peripherally inserted central catheter [All fields]

#6 PICC [All fields]

#7 catheterization, central venous [MeSH terms]

#8 OR #5-7

#9 pediatric [All fields]

#10 children [All fields]

#11 infant [All fields]

#12 neonate [All fields]

#13 child [MeSH terms]

#14 infant, newborn [MeSH terms]

#15 OR #9-14

#16 efficacy [All fields]

#17 safety [All fields]

#18 treatment outcome [MeSH terms]

#19 OR #15-18

#20 #4 AND #8 AND #15 AND #19

**For the Cochrane databases**

ID Search

#1 "intracavitary electrocardiography"

#2 “IC-ECG"

#3 "electrocardiography, intracavitary"

#4 OR #1-3

#5 "peripherally inserted central catheter"

#6 "PICC"

#7 "central venous catheterization"

#8 OR #5-7

#9 "pediatric"

#10 "children"

#11 “infant”

#12 “neonate”

#13 OR #9-12

#14 "efficacy"

#15 "safety"

#16 "treatment outcome"

#17 OR #13-16

#18 #4 AND #8 AND #13 AND #17

**For Embase databases**

('intracavitary electrocardiography':ti,ab OR 'ic-ecg':ti,ab) AND ('peripherally inserted central catheter':ti,ab OR 'picc':ti,ab) AND ('pediatric':ti,ab OR 'children':ti,ab OR 'infant':ti,ab OR ' neonate':ti,ab ) AND ('efficacy':ti,ab OR 'safety':ti,ab)
