# Supplementary material for: Efficacy and safety of intracavitary electrocardiography-guided peripherally inserted central catheters in pediatric patients: a systematic review and meta-analysis
Source: PeerJ. 2024 Oct 8;12:e18274. doi: 10.7717/peerj.18274 (PMC11468838; doi:10.7717/peerj.18274)
Supplement: Supplemental Information 5 [file peerj-12-18274-s005.docx]

**Table S1. JBI critical appraisal tool for the assessment of risk of bias quasi-experimental studies.**

| Author,year | Q1 | Q2 | Q3 | Q4 | Q5 | Q6 | Q7 | Q8 | Q9 | Q10 |
| --- | --- | --- | --- | --- | --- | --- | --- | --- | --- | --- |
| Capasso et al.2018 | Y | N | Y | N | Y | Y | Y | Y | Y | Y |
| D’Andrea et al.2022 | Y | N | Y | N | Y | Y | Y | Y | Y | Y |
| Raffaele et al.2020 | Y | N | Y | U | U | Y | Y | U | Y | Y |
| Yang et al.2019 | Y | N | Y | N | Y | Y | Y | Y | Y | Y |
| Zhang et al.2022 | Y | N | Y | N | Y | Y | Y | Y | Y | Y |
| Zhou et al.2017 | Y | Y | Y | Y | Y | Y | Y | Y | Y | Y |

Q1. Is it clear in the study what is the “cause” and what is the “effect” (i.e. there is no confusion about which variable comes first)?;Q2. Was there a control group?;Q3. Were participants included in any comparisons similar?;Q4.Were the participants included in any comparisons receiving similar treatment/care, other than the exposure or intervention of interest?;Q5. Were there multiple measurements of the outcome, both pre and post the intervention/exposure?;Q6. Were the outcomes of participants included in any comparisons measured in the same way?;Q7. Were outcomes measured in a reliable way?; Q8.Was follow-up complete and if not, were differences between groups in terms of their follow-up adequately described and analyzed?;Q9. Was appropriate statistical analysis used?;Q10.Is it included?.

Y.Yes; N.NO; U.Unclear.
