# Supplementary material for: Efficacy and safety of intracavitary electrocardiography-guided peripherally inserted central catheters in pediatric patients: a systematic review and meta-analysis
Source: PeerJ. 2024 Oct 8;12:e18274. doi: 10.7717/peerj.18274 (PMC11468838; doi:10.7717/peerj.18274)
Supplement: Supplemental Information 6 [file peerj-12-18274-s006.zip › Supplementary Table 3.docx]

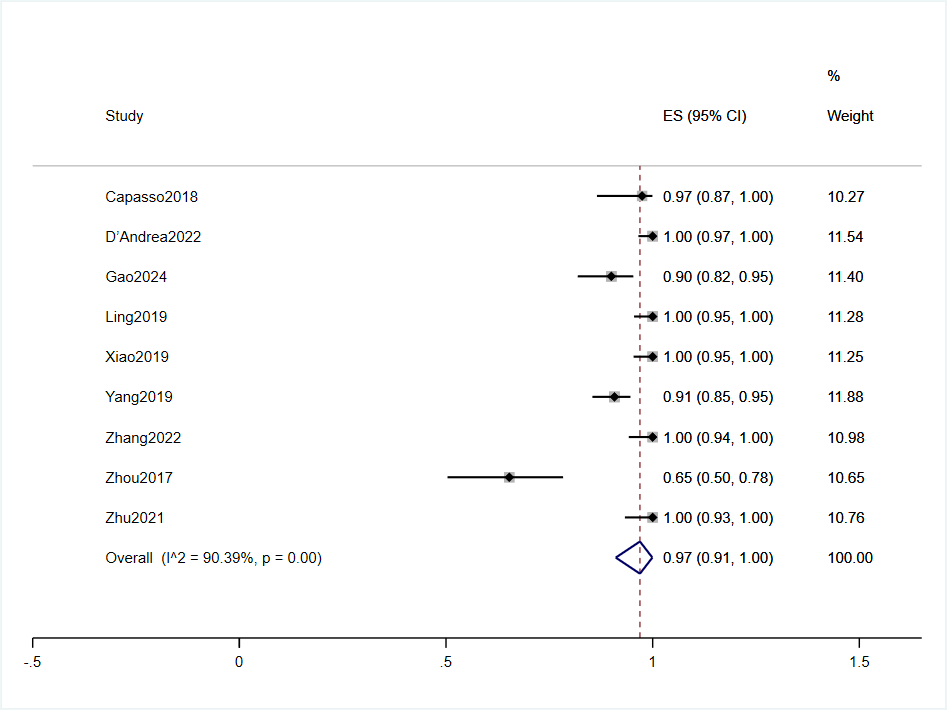
Fig.S3.1: Pooled analysis of applicability.


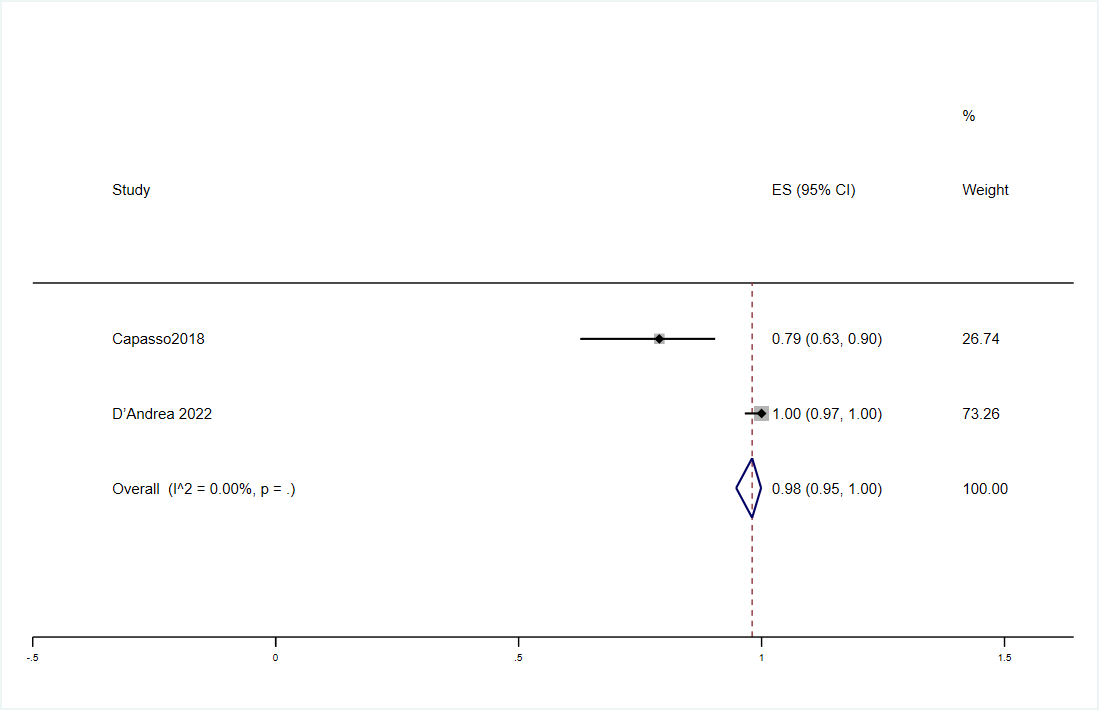
Fig.S3.2: Pooled analysis of feasibility.


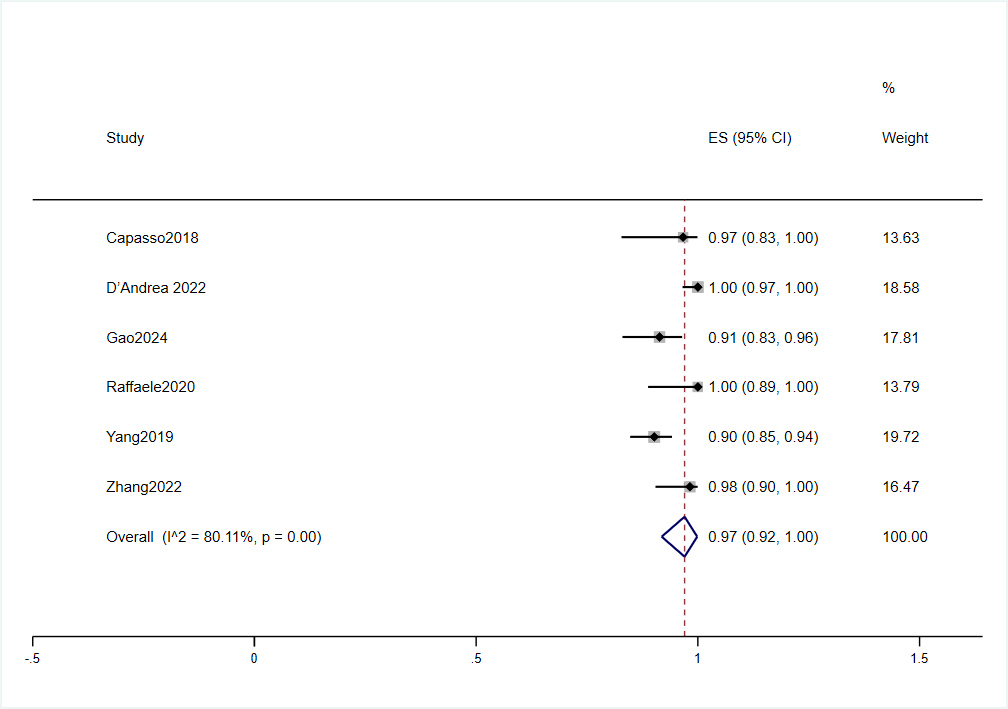
Fig.S3.3: Pooled analysis of the accuracy for puncture.


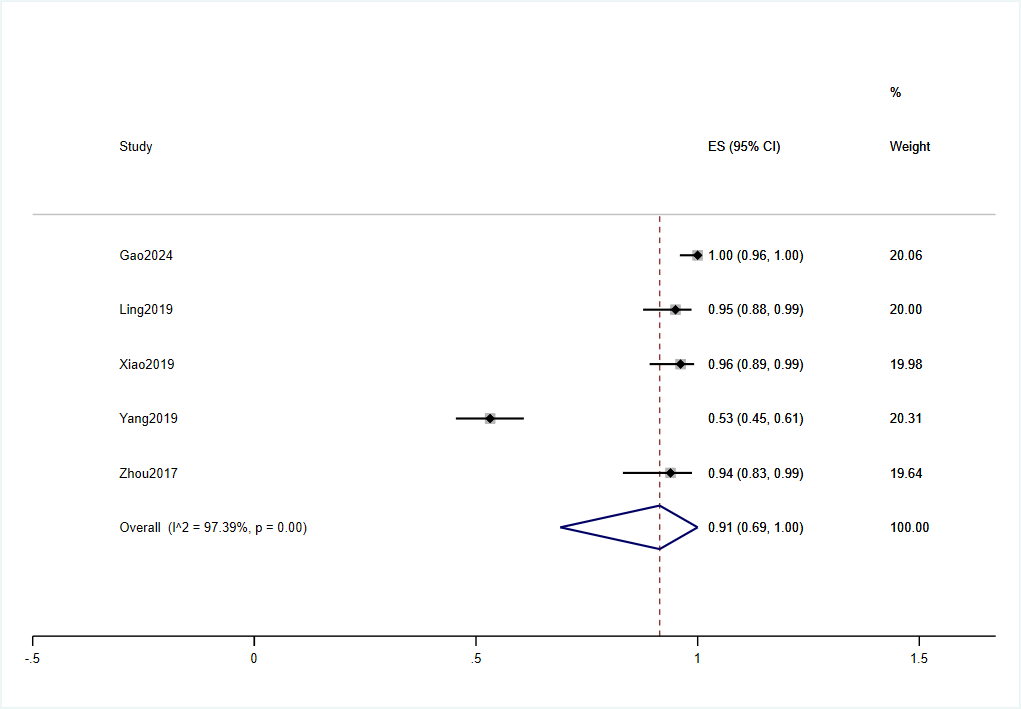
Fig.S3.4: Pooled analysis of the first puncture success rate.


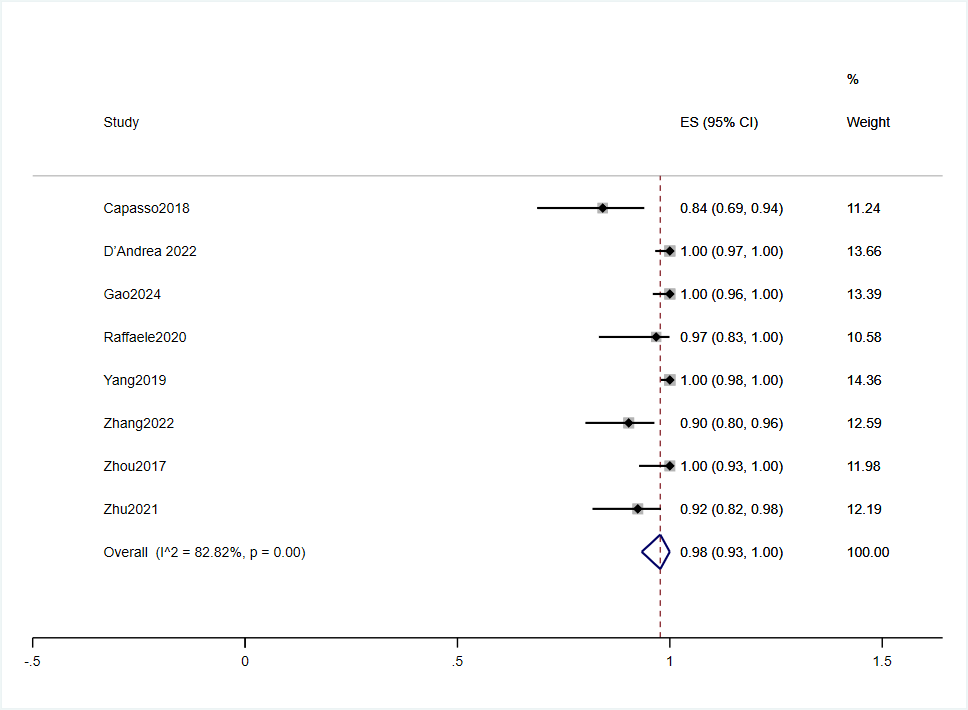
Fig.S3.5: Pooled analysis of overall success rate.


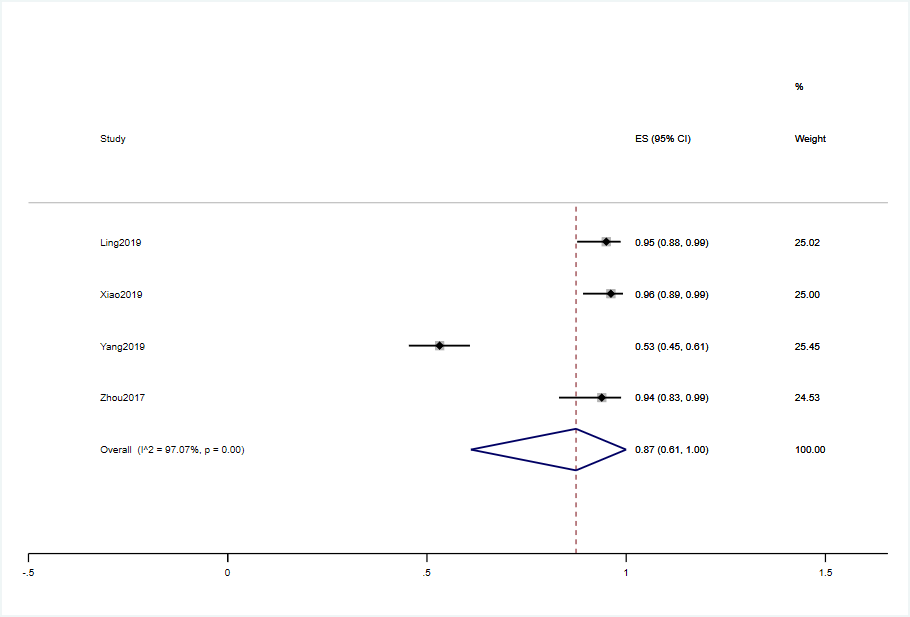
Fig.S3.6: Pooled analysis of the first puncture success rate in neonates.


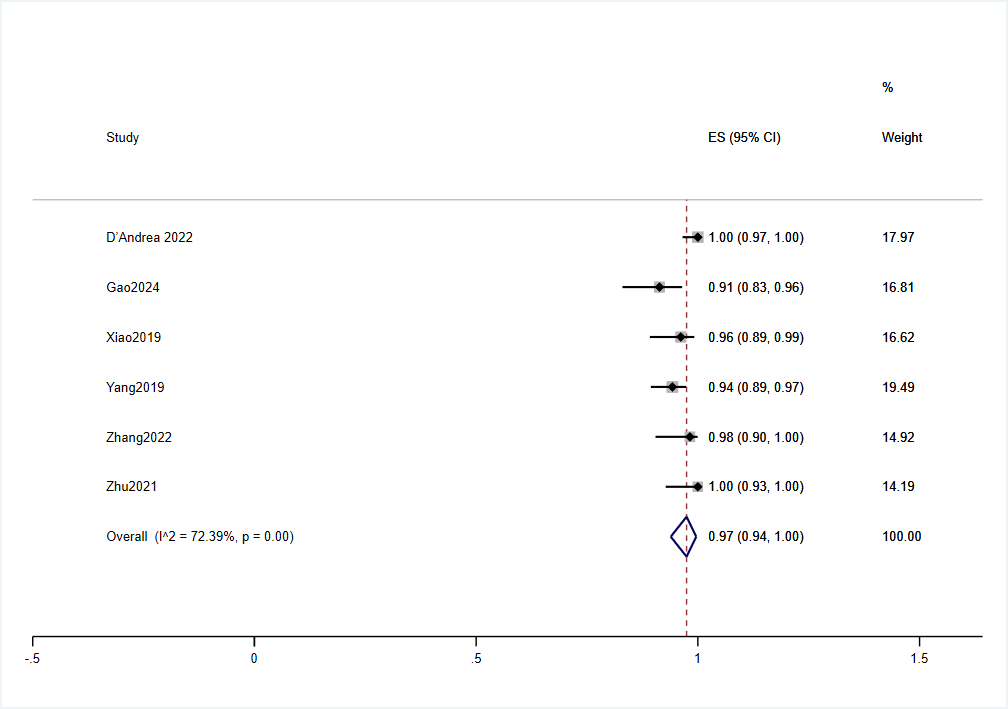
Fig.S3.7: Pooled analysis of sensitivity.


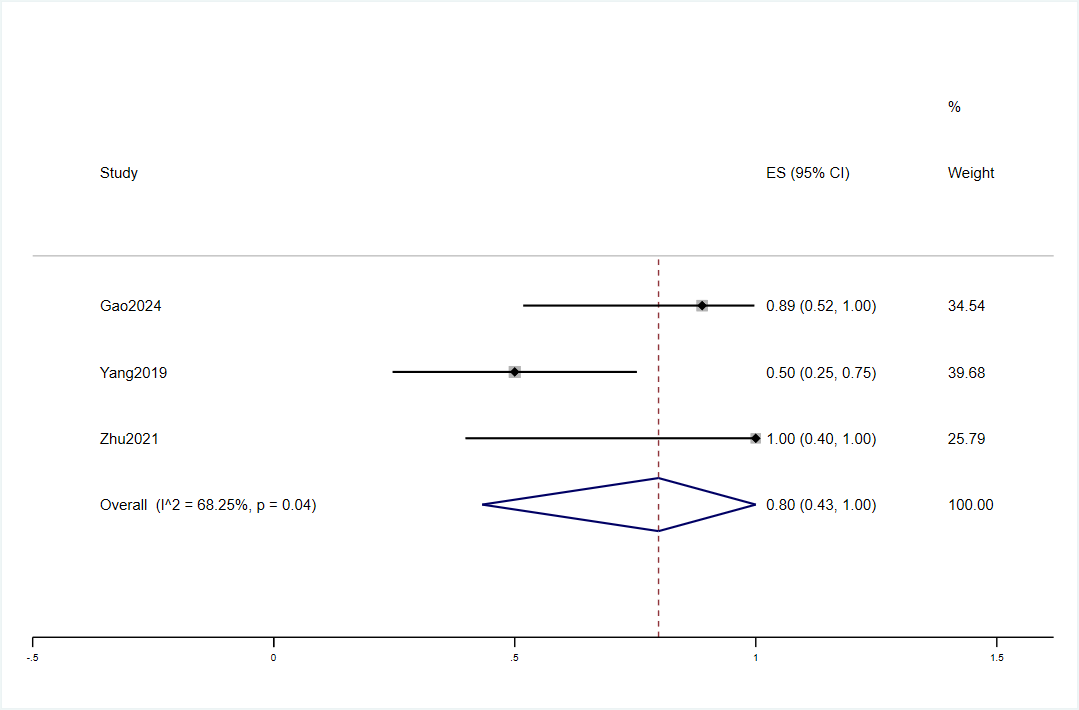
Fig.S3.8: Pooled analysis of specificity.


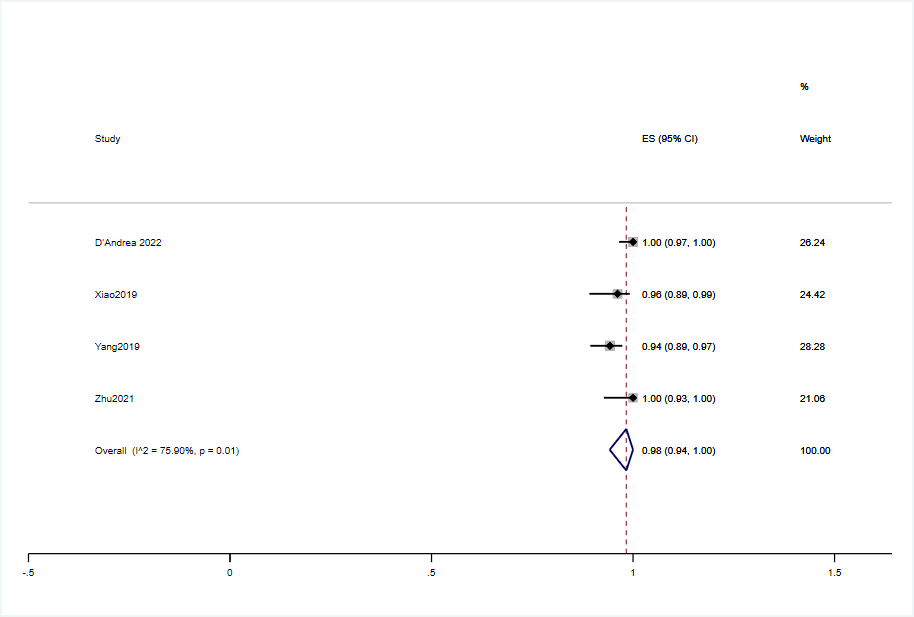


Fig.S3.9: Pooled analysis of sensitivity in neonates.


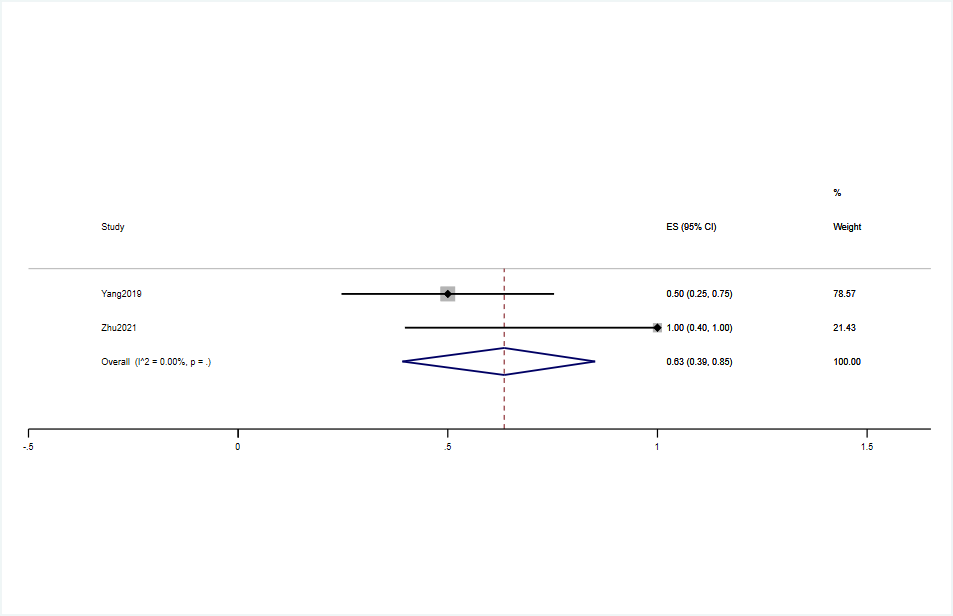
Fig.S3.10: Pooled analysis of specificity in neonates.


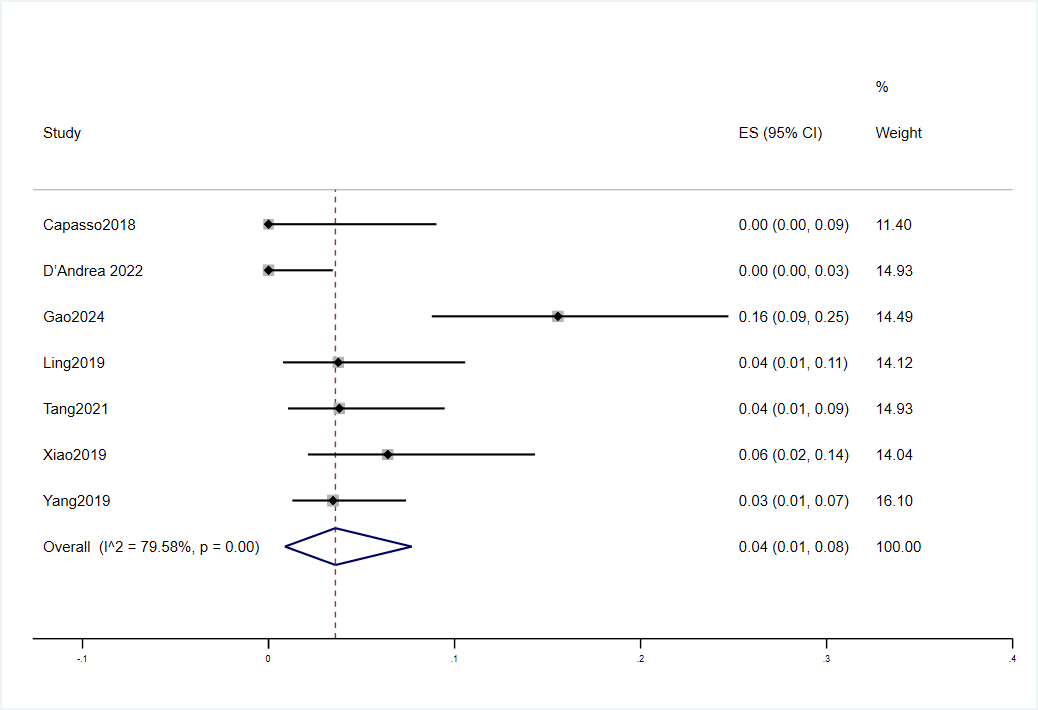


Fig.S3.11: Pooled analysis of overall complication.


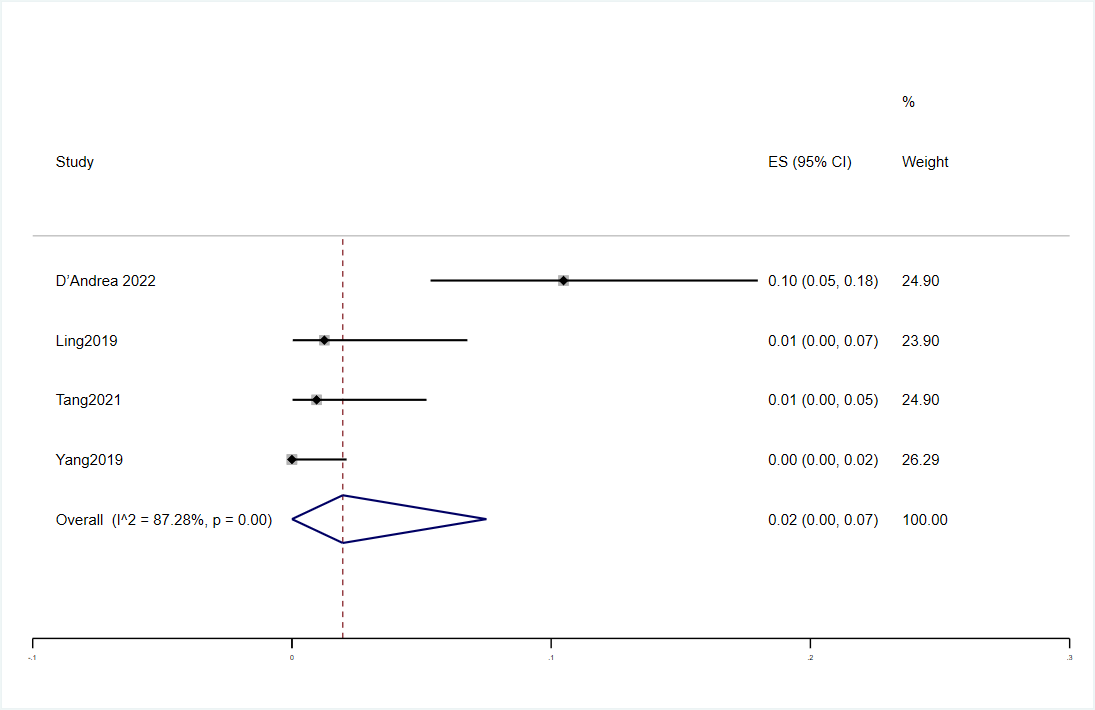
Fig.S3.12: Pooled analysis of catheter site inflammation.


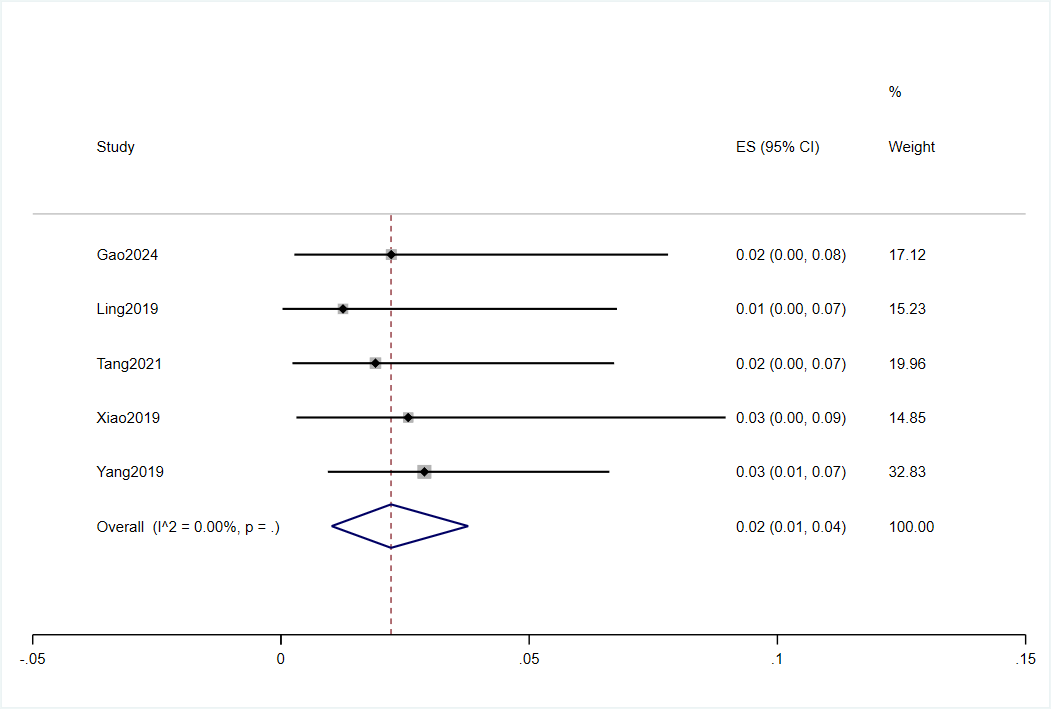
Fig.S3.13: Pooled analysis of phlebitis.


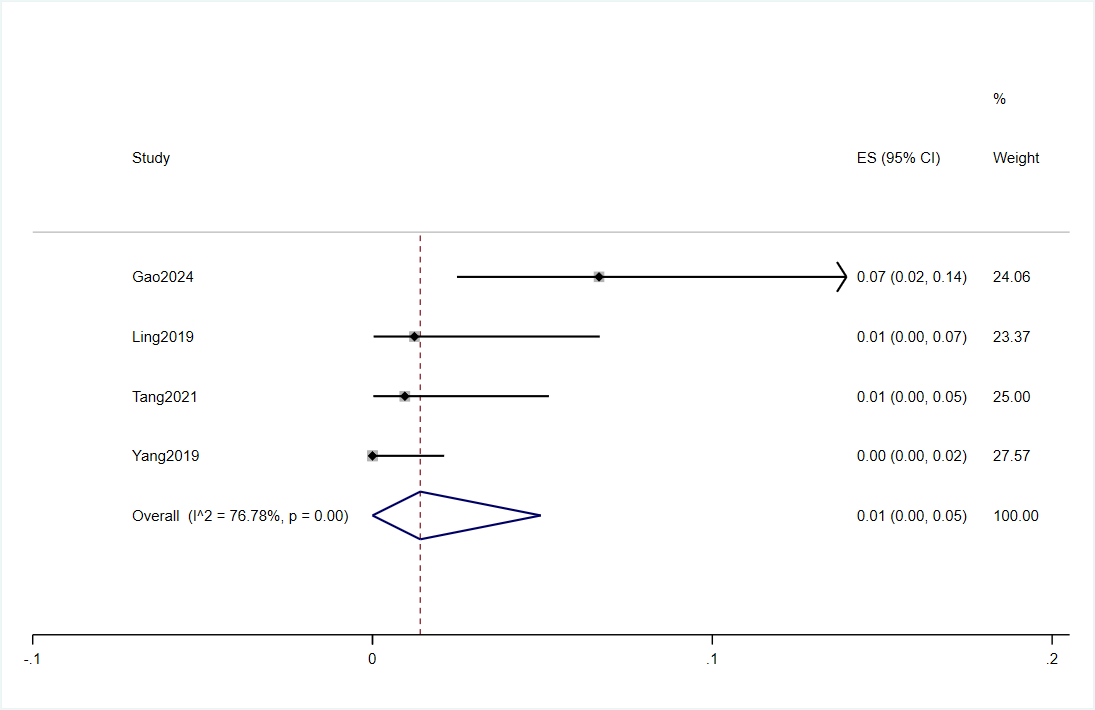


Fig.S3.14: Pooled analysis of thrombosis.


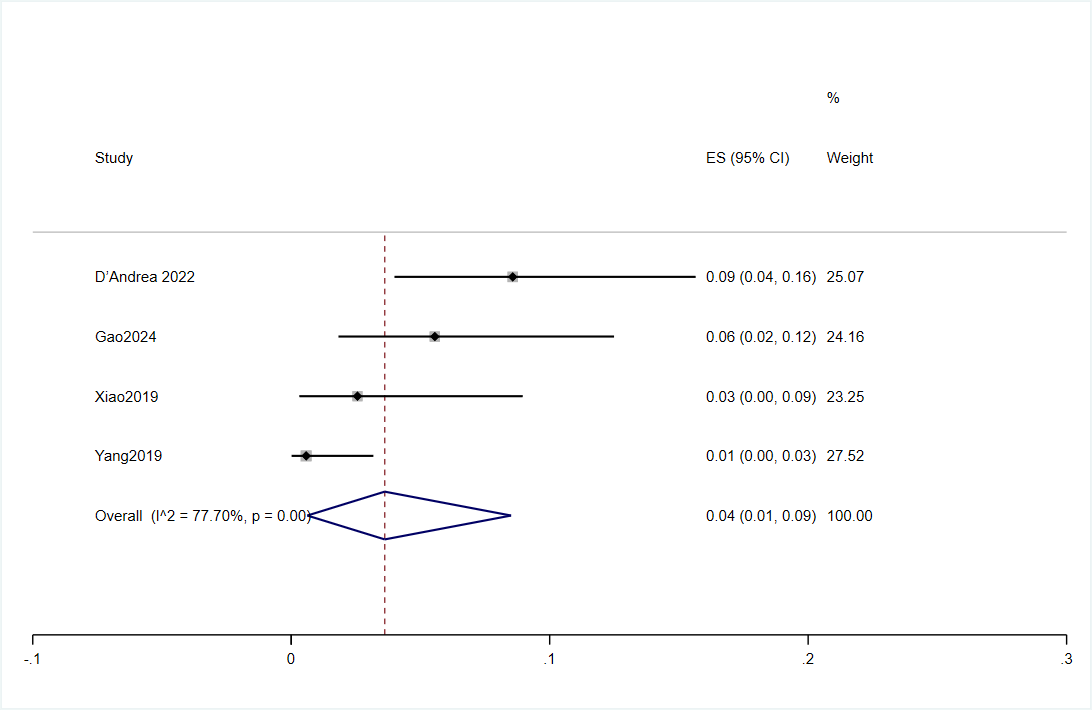


Fig.S3.15: Pooled analysis of catheter malposition.
