# Supplementary material for: Efficacy and safety of intracavitary electrocardiography-guided peripherally inserted central catheters in pediatric patients: a systematic review and meta-analysis
Source: PeerJ. 2024 Oct 8;12:e18274. doi: 10.7717/peerj.18274 (PMC11468838; doi:10.7717/peerj.18274)
Supplement: Supplemental Information 8 [file peerj-12-18274-s008.docx]

**Rationale:**

This systematic review and meta-analysis was conducted to address the limited systematic evidence available on the efficacy and safety of Intracavitary Electrocardiography (IC-ECG) guided Peripherally Inserted Central Catheters (PICC) placement in pediatric patients, including neonates. Given the unique anatomical and physiological challenges in pediatric patients, there is a significant need to evaluate methods that reduce procedural complications and improve clinical outcomes. IC-ECG offers a promising alternative to traditional radiographic methods, potentially reducing radiation exposure and improving placement accuracy.

**Contribution to Knowledge:**

This work contributes significantly to existing literature by providing a comprehensive, up-to-date synthesis of evidence comparing IC-ECG guidance to traditional methods in pediatric settings. Unlike previous reviews, which either focus broadly on adult populations or do not systematically assess the pediatric-specific outcomes, this study rigorously evaluates the appropriateness, safety, and efficacy of IC-ECG in children and neonates. The findings offer clear, statistically significant evidence supporting the superior performance of IC-ECG in terms of placement success rates and reduced complications. Moreover, this analysis is one of the first to incorporate recent high-quality randomized controlled trials, thereby providing the most robust conclusions to date on this topic and guiding future clinical practices and policies.
